# Evaluating the Role of Breast Ultrasound in Early Detection of Breast Cancer in Low- and Middle-Income Countries: A Comprehensive Narrative Review

**DOI:** 10.3390/bioengineering11030262

**Published:** 2024-03-07

**Authors:** Roxana Iacob, Emil Radu Iacob, Emil Robert Stoicescu, Delius Mario Ghenciu, Daiana Marina Cocolea, Amalia Constantinescu, Laura Andreea Ghenciu, Diana Luminita Manolescu

**Affiliations:** 1 Department of Anatomy and Embriology, ‘Victor Babeș’ University of Medicine and Pharmacy, 300041 Timișoara, Romania; roxana.iacob@umft.ro; 2 Doctoral School, ‘Victor Babeș’ University of Medicine and Pharmacy, 300041 Timișoara, Romania; stoicescu.emil@umft.ro (E.R.S.); mario.ghenciu@umft.ro (D.M.G.); daiana.cocolea@umft.ro (D.M.C.); 3 Faculty of Mechanics, Field of Applied Engineering Sciences, Specialization Statistical Methods and Techniques in Health and Clinical Research, ‘Politehnica’ University Timișoara, Mihai Viteazul Boulevard No. 1, 300222 Timisoara, Romania; 4 Department of Pediatric Surgery, ‘Victor Babeș’ University of Medicine and Pharmacy, 300041 Timișoara, Romania; 5 Department of Radiology and Medical Imaging, ‘Victor Babeș’ University of Medicine and Pharmacy, 300041 Timișoara, Romania; amalia.constantinescu@umft.ro (A.C.); dmanolescu@umft.ro (D.L.M.); 6 Research Center for Pharmaco-Toxicological Evaluations, ‘Victor Babeș’ University of Medicine and Pharmacy, 300041 Timișoara, Romania; 7 Discipline of Pathophysiology, ‘Victor Babeș’ University of Medicine and Pharmacy, 300041 Timișoara, Romania; bolintineanu.laura@umft.ro; 8 Center for Research and Innovation in Precision Medicine of Respiratory Diseases (CRIPMRD), ‘Victor Babeș’ University of Medicine and Pharmacy, 300041 Timișoara, Romania

**Keywords:** breast cancer, mammography, breast ultrasound, automatic breast ultrasound, breast cancer screening, breast cancer imaging, breast cancer imaging methods, cancer in low income countries, cancer in middle-income countries, breast cancer management

## Abstract

**Simple Summary:**

This review analyzes the possibility of breast ultrasound as a primary screening modality for breast cancer, particularly in resource-limited areas. It examines 52 recent papers and underlines ultrasound’s advantages, including radiation-free imaging and suitability for younger populations. Reduced specificity and operator reliance are two major challenges. Despite advances such as automatic breast ultrasound (ABUS), this review emphasizes the importance of a comprehensive screening approach, with a focus on international collaboration to enhance global outcomes.

**Abstract:**

Breast cancer, affecting both genders, but mostly females, exhibits shifting demographic patterns, with an increasing incidence in younger age groups. Early identification through mammography, clinical examinations, and breast self-exams enhances treatment efficacy, but challenges persist in low- and medium-income countries due to limited imaging resources. This review assesses the feasibility of employing breast ultrasound as the primary breast cancer screening method, particularly in resource-constrained regions. Following the PRISMA guidelines, this study examines 52 publications from the last five years. Breast ultrasound, distinct from mammography, offers advantages like radiation-free imaging, suitability for repeated screenings, and preference for younger populations. Real-time imaging and dense breast tissue evaluation enhance sensitivity, accessibility, and cost-effectiveness. However, limitations include reduced specificity, operator dependence, and challenges in detecting microcalcifications. Automatic breast ultrasound (ABUS) addresses some issues but faces constraints like potential inaccuracies and limited microcalcification detection. The analysis underscores the need for a comprehensive approach to breast cancer screening, emphasizing international collaboration and addressing limitations, especially in resource-constrained settings. Despite advancements, notably with ABUS, the primary goal is to contribute insights for optimizing breast cancer screening globally, improving outcomes, and mitigating the impact of this debilitating disease.

## 1. Introduction

Breast cancer is a highly prevalent form of cancer that affects both genders, although it is more commonly found in women [1]. While the precise etiology of breast cancer remains incompletely elucidated, several risk factors including older age, familial predisposition, hormonal effects, and specific genetic abnormalities have been found [2]. Breast cancer has the potential to occur at any point of life; however, it is frequently detected in women who are 40 years of age or older. Nevertheless, it is crucial to acknowledge that younger women and, on rare occasions, men might also experience the same impacts [3,4]. Regular breast self-examinations, clinical breast examinations, and mammography serve as essential methods for early detection, greatly enhancing the likelihood of effective therapy [5,6]. Early-stage breast cancer frequently manifests without apparent symptoms, underscoring the crucial significance of screening in detecting the illness within its earlier, less severe stages [6].

Breast cancer presents a wide variety of subtypes, and its occurrence often shows an intricate correlation with age. Traditionally, postmenopausal women were regarded as being at an elevated risk, as the occurrence of breast cancer progressively increases with age. Nevertheless, there has been a discernible change in recent years, demonstrating a higher incidence of breast cancer among younger demographics [7]. The increase in this phenomenon can be attributed to various variables, such as changes in lifestyle, a delay of childbirth, the utilization of hormonal contraceptives, and an increase in genetic predispositions [8,9]. Recent statistics indicate that younger women are currently being diagnosed with different subtypes of breast cancer, including the triple-negative and HER2-positive subtypes, which are known to have a higher level of aggressiveness [3,10]. Furthermore, increased consciousness, more accurate screening techniques, and improved diagnostic abilities may have roles in identifying breast cancer at earlier stages, including in younger women [3]. As the awareness of the complex relationship between age, genetics, and environmental variables improves, the changing patterns of breast cancer demographics highlight the significance of customized screening and prevention approaches for different age cohorts. Ongoing research and increased awareness are crucial to fully understand and effectively respond to the evolving patterns of breast cancer occurrence and characteristics.

The symptoms of breast cancer can be varied, and certain individuals might not experience any symptoms during the initial stages [11]. Typical signs consist of the existence of a mass or thickening in the breast or armpits; modifications in the dimensions, form, or visual aspect of the breast; changes in the nipple, such as turning inward or leaking fluid; and alterations in the skin, such as redness or the formation of small depressions. Regular screenings and awareness initiatives have the objective of educating individuals about these indicators, promoting a rapid intervention [12,13].

Diverse imaging modalities are crucial in the process of screening and diagnosing breast cancer. Mammography, the main diagnostic technique, captures high-resolution X-ray pictures, identifying both masses and microcalcifications. Enhanced image quality in digital mammography facilitates efficient diagnoses. Breast ultrasonography, often used alongside mammography, provides imaging without ionizing radiation, making it particularly effective in analyzing breast masses and assessing dense tissue [14]. Magnetic resonance imaging (MRI) utilizes magnets and electromagnetic radiation to produce precise and detailed images, making it particularly advantageous in situations with high-risk patients [15]. Mammography continues to be the main technique used, although breast ultrasonography plays a valuable part in a complete and patient-friendly approach to breast health. It is safe and effective in defining masses and can be used in cases of dense breast tissue [16,17].

The availability of imaging techniques in low- and medium-income countries continues to be a major obstacle, impeding the effectiveness of cancer screening and diagnosis. Insufficient funding and limitations in infrastructure frequently lead to a shortage of advanced imaging technologies, such as mammography and magnetic resonance imaging (MRI), which are essential for the early detection of cancer and precise diagnosis [16,18,19]. In these places, where healthcare budgets are under pressure and the need to prioritize is crucial, the expenses associated with obtaining and upkeeping cutting-edge imaging equipment become unaffordable. The limited availability of these resources hinders the capacity to execute comprehensive cancer screening initiatives and delays prompt and precise diagnoses. Consequently, people in low- and medium-income nations may experience a delay in the detection of malignancies, resulting in the diagnosis of more advanced stages, limited treatment choices, and ultimately worse results. It is essential to prioritize the resolution of the inequality in the availability of imaging technologies in order to build cancer control programs that are successful and enhance the overall health outcomes in underserved locations. International cooperation between organizations, governments, and non-governmental entities is crucial to narrow the divide and guarantee fair access to crucial imaging resources for cancer screening and diagnosis in countries with lower and moderate incomes [19].

The continuous progress in imaging technology always expands the limits of diagnostic precision, introducing new eras of accuracy and effectiveness in healthcare. Automatic breast ultrasound (ABUS) is a revolutionary advancement in breast imaging that significantly transforms the field of diagnostic ultrasound procedures [20,21]. ABUS, in contrast to conventional handheld ultrasonography, is distinguished by its automated scanning procedure, which employs a specialized machine to capture a sequence of standardized images of the entire breast. This technique guarantees comprehensive coverage and uniform imaging, effectively tackling the difficulties related to operator reliance and fluctuations in handheld ultrasound exams. ABUS provides a methodical approach to breast imaging, improving the consistency of outcomes and reducing the chances of omitting any potential changes. This technology offers significant benefits in screening situations, allowing for a thorough assessment of breast tissue without being limited by the expertise of the operator. This automated technology not only speeds up the imaging process but also enhances the consistency and unbiased evaluation, enabling better identification of anomalies and potentially decreasing the inherent subjectivity in traditional handheld ultrasound tests [22,23]. The introduction of ABUS marks the beginning of a hopeful period in breast health diagnostics, providing a dependable and effective option that complements and improves the capabilities of handheld ultrasound in the pursuit of early breast cancer diagnosis [24].

The major goal of this narrative review is to analyze the feasibility of using breast ultrasound as the primary screening method for breast cancer, particularly in low- and middle-income locations with limited resources and access to modern medical facilities. Specifically, we want to discover the constraints and conditions under which breast ultrasonography can be used effectively in breast cancer screening in these resource-constrained settings. By focusing on the assessment of restrictions and examining different implementation scenarios, we hope to provide insights into the feasibility and viability of employing breast ultrasound as an alternative and cost-effective screening technique in such situations.

Estimating the extent of limited resources and restricted availability of advanced medical facilities in low- and middle-income countries (LMICs) is challenging but widely recognized. Reports from organizations like the World Health Organization (WHO) and the International Agency for Research on Cancer (IARC) highlight significant barriers to adequate cancer screening and diagnosis in these regions. Challenges include infrastructural deficiencies, shortages of trained healthcare professionals, and financial constraints. While precise estimates vary, it is evident that LMICs face substantial obstacles in providing essential cancer services, leading to disparities in cancer outcomes compared to high-income countries. Addressing these challenges requires investment in healthcare infrastructure and the development of cost-effective screening strategies tailored to local contexts.

By evaluating the benefits and constraints of breast ultrasonography, our objective is to offer an understanding into its potential as a practical and easily available method for the early diagnosis of breast cancer in situations where mammography or magnetic resonance imaging may present logistical and budgetary obstacles. This study aims to provide significant insights into the ongoing initiatives focused on refining breast cancer screening strategies, with the ultimate objective of improving outcomes and reducing the impact of this devastating disease in resource-limited settings.

## 2. Materials and Methods

The PRISMA (Preferred Reporting Items for Systematic Reviews and Meta-Analyses) standards were followed in the selection of the studies that were included in this research [25]. The current literature review is based on bibliographic searches conducted using MeSH terms (on PubMed) and both manual and automated searches in the PubMed database, Google Scholar, and Scopus. The latest six-year period of publications on ultrasound and ABUS application in screening and diagnosis of breast cancer was chosen. Based on their title, the details provided in their abstract, and a brief look at the entire paper, the most relevant articles were selected. We eliminated publications with only the abstract available, duplicates, and articles published in languages other than English. Also, articles that were not considered relevant for our aim were excluded.

Three radiologists with expertise in senology carried out the search and careful selection of the articles in December 2023. Initially, the research papers were hand-searched in the above-mentioned databases, using the following keywords “breast ultrasound in breast cancer screening”, “automatic breast ultrasound in cancer screening”, “breast ultrasound in breast cancer screening”, and “automatic breast ultrasound in cancer diagnosis”. Afterwards, we performed a second search using the MeSH term option that is available in PubMed, with the following terms:

((“Breast Neoplasms”[Mesh]) AND “Ultrasonography, Mammary”[Mesh]) AND “Diagnosis”[Mesh];
((“Ultrasonography, Mammary”[Mesh]) AND “Early Detection of Cancer”[Mesh]) AND “Breast Neoplasms”[Mesh];
((“Breast Neoplasms”[Mesh]) AND “Ultrasonography, Mammary”[Mesh]) AND “Diagnosis”[Mesh];
((“Breast Neoplasms”[Mesh]) AND “Mass Screening”[Mesh]) AND “Ultrasonography”[Mesh].

All of the articles selected were added to a Microsoft Excel table with the following columns for improved management and organization of the review: title, authors, year and journal of publication, type of publication, keywords, and advantages and/or disadvantages of ultrasound/ABUS.

We chose the most relevant publications based on their advantages and benefits, as well as the researchers’ findings about the method’s limitations. Finally, 52 articles were chosen for the literature review since they fulfilled all the requirements. We talked about their key findings and organized the outcomes as follows:

- Overall advantages of using breast ultrasound for breast tissue evaluation;
- Advantages of breast ultrasound in breast cancer screening;
- Disadvantages/limitations of breast ultrasound in breast cancer screening;
- Advantages of breast ultrasound in breast cancer diagnosis;
- Disadvantages/limitations of breast ultrasound in breast cancer diagnosis;
- ABUS in breast cancer screening and diagnosis—advantages and limitations.

**Figure 1:**
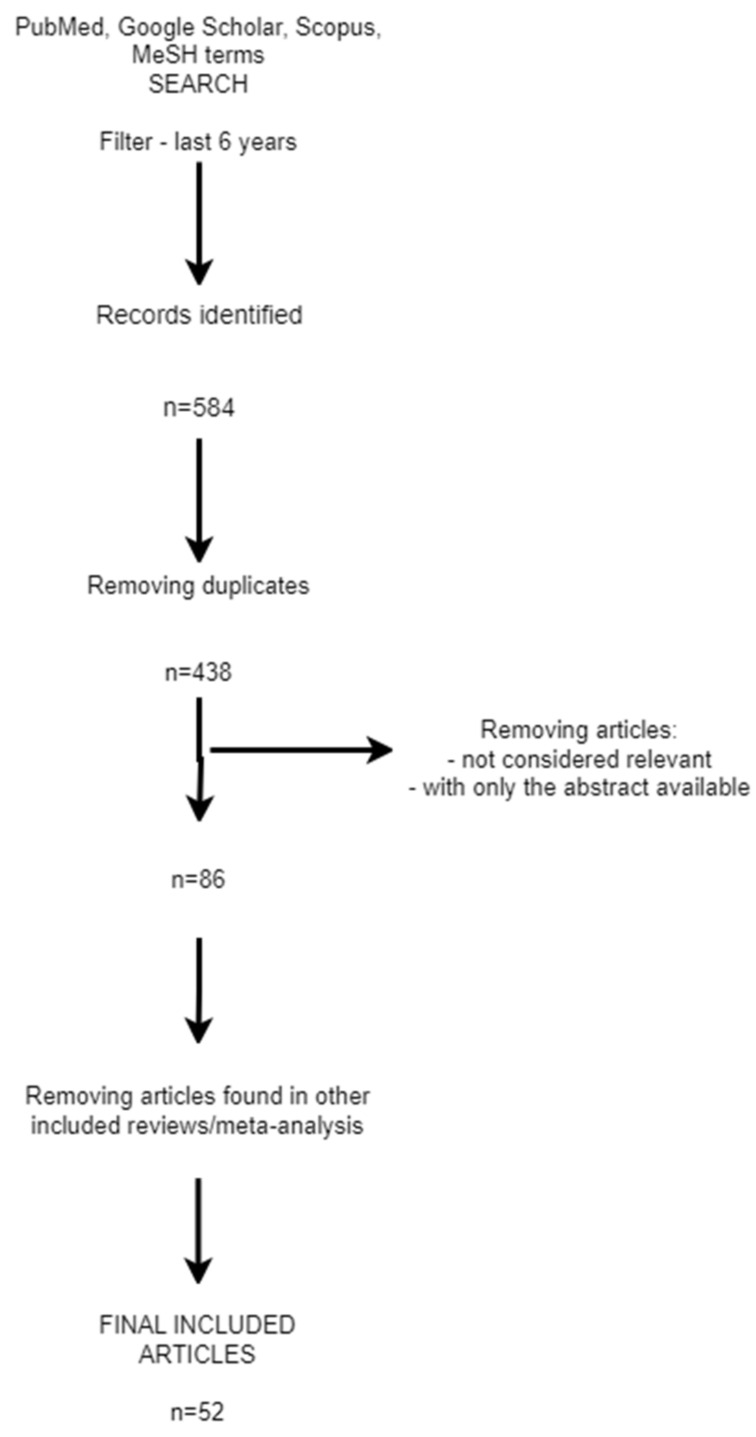
PRISMA diagram.

The process followed for selecting the articles for the review is summarized in the diagram below—a PRISMA diagram (Figure 1).

The literature review followed the PRISMA guidelines and focused on ultrasound and ABUS in breast cancer screening and diagnosis. PubMed, Google Scholar, and Scopus searches were searched using MeSH terms, with a focus on publications published within the last six years. Three radiologists reviewed the 52 pertinent papers, emphasizing their benefits and limitations. The review emphasized the necessity of understanding the capabilities of ultrasound and ABUS in breast cancer management in order to optimize screening techniques and improve patient outcomes.

**Figure 2:**
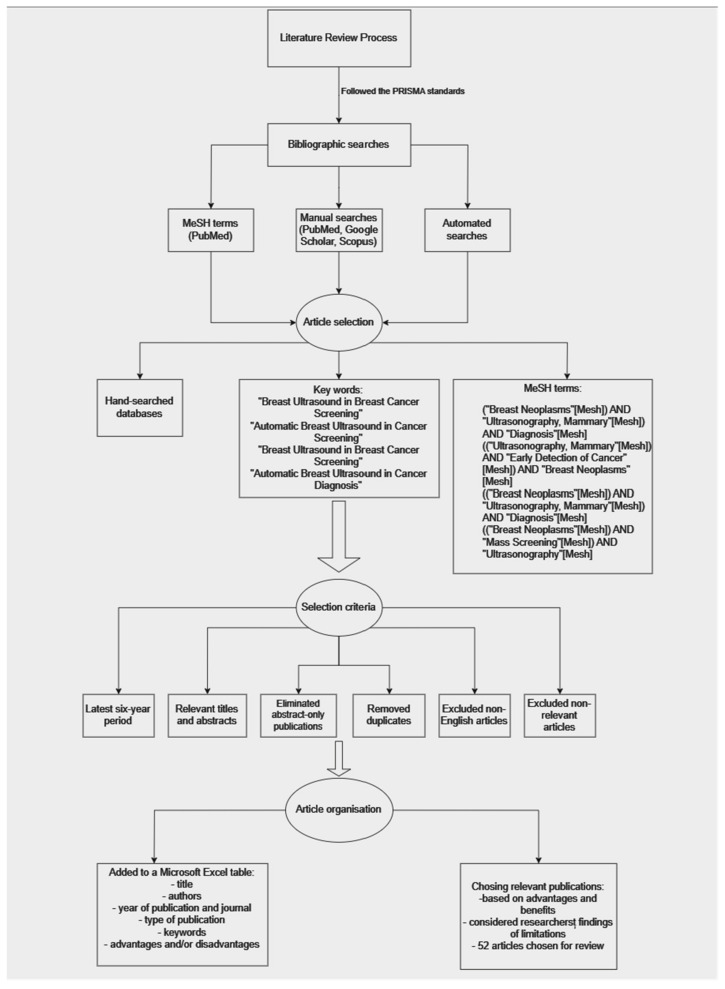
Tree diagram of the conducted research.

The following tree diagram illustrates the systematic process of conducting the literature review, from following the PRISMA standards to selecting and organizing relevant articles for the review (Figure 2).

## 3. Overall Advantages of Using Breast Ultrasound for Breast Tissue Evaluation

### 3.1. Absence of Ionizing Radiation

An important benefit of breast ultrasonography is its utilization of sound waves instead of ionizing radiation, which is a fundamental element of mammography. Mammograms employ X-rays to generate precise images of the breast tissue. Although they have demonstrated efficacy, the exposure to ionizing radiation gives rise to concerns, particularly over its cumulative impact during repeated screenings and at a younger age, and in pregnant women. Unlike other imaging techniques, breast ultrasound utilizes benign sound waves, eliminating the potential hazards of radiation. This renders it a safer option for individuals who may necessitate regular screenings or possess heightened susceptibility to radiation exposure [13,22,23].

### 3.2. Suitability for Repeated Screenings

The lack of ionizing radiation in breast ultrasonography makes it very suitable for repeated scans over an extended period. Individuals who undertake regular surveillance, such as those with a family history of breast cancer or genetic predispositions, may be concerned about the cumulative effects of radiation from repeated mammograms. Utilizing breast ultrasonography as an alternative eliminates the need for radiation, enabling more regular exams without the accompanying hazards. This is especially beneficial in situations where timely and consistent monitoring is crucial, providing a more secure and sustainable option for persons in need of continuous breast imaging evaluations [18,26,27].

### 3.3. Favorable Choice for Younger Patients

Persons in younger age groups, particularly females below 40 years old, might be more vulnerable to the possible lifelong consequences of ionizing radiation. This particular group, which tends to prioritize breast health and preventative measures, may perceive breast ultrasonography as a preferable option for screening. The safety of ultrasound is in line with the preferences and concerns of younger individuals, making it an attractive choice for those who want to begin monitoring their breast health at a younger age without jeopardizing their long-term welfare [19,28,29].

### 3.4. Psychological Impact for Patients

The patient’s experience with breast ultrasonography extends beyond the therapeutic domain, incorporating elements like as comfort, decreased fear, and the possibility of point-of-care applications. Contrary to other imaging methods that can cause discomfort or compression as well, breast ultrasonography is a gentle technique that typically involves the application of a gel and the utilization of a handheld device on the skin. This tactile and delicate approach enhances the comfort of the experience, reducing the anxiety that certain individuals may associate with breast imaging. Moreover, ultrasound results offer a more expedited processing time in comparison to mammography, providing early data to the referring physician and reducing patient anxiety by enabling speedier evaluations and timely medical knowledge [30,31,32].

## 4. Advantages of Breast Ultrasound in Breast Cancer Screening

Recent studies and articles highlight several advantages of breast ultrasound in the context of breast cancer screening, and while it may not replace mammography entirely, it demonstrates considerable reliability and effectiveness when used alone.

### 4.1. Enhanced Sensitivity, Particularly in Dense Breasts

Recent research emphasizes the increased sensitivity of breast ultrasonography, particularly in women with dense breast tissue, where mammography may have reduced effectiveness. Mammograms may produce false negative results due to the presence of dense breast tissue, which can mask abnormalities. Utilized as an adjunctive screening modality, breast ultrasonography has demonstrated notable advantages in identifying extra malignancies that may escape detection with mammography alone. Recent research has indicated substantial enhancements in overall sensitivity, hence decreasing the probability of undiscovered malignancies in women with thick breasts [17,33,34,35,36,37,38,39,40,41]. While one study suggests that the sensitivity and specificity of ultrasound, compared to the histopathological results, are at 85.8%, and 73.3%, respectively [41], another study compared the accuracy of ultrasound and mammography in terms of lesion’s dimensions. Thus, it was demonstrated that the specificity is higher for both of the methods (78.7% for mammography and 92.9% for ultrasound), when the lesion is between 1.1 and 2 cm, compared to the specificities for lesions smaller than 1 cm (65.2% for mammography and 85.1% for ultrasound [42].

### 4.2. Versatility and Dynamic Real-Time Imaging

Breast ultrasound enables the evaluation of breast tissue through dynamic and real-time imaging. The ability to adapt and perform well when identifying minor irregularities and evaluating the features of lesions is a valuable asset that aids in making precise diagnoses. This real-time feature enables the use of ultrasound guidance during biopsies, which improves the accuracy of tissue sample and reduces the necessity for more invasive treatments [16,43,44].

### 4.3. Accessibility and Cost-Effectiveness

Recent research highlights the ease of use and cost efficiency of breast ultrasonography, making it an appealing option for a variety of medical environments. Ultrasound machines are comparatively more cost-effective and portable than mammography or magnetic resonance imaging (MRI) machines, considering their initial price and infrastructure requirements. The price of breast ultrasonography improves the practicality of including it in screening programs, particularly in places with low resources, hence guaranteeing wider availability of early detection techniques [16,18,19,26,45].

### 4.4. Application in High-Risk Populations and Younger Women

Screening high-risk populations and younger women has been shown to be particularly effective with the use of breast ultrasonography. Recent studies suggest that it is useful in identifying cancerous tumors in these populations, especially when mammography may not be as effective. Ultrasound’s capacity to detect lesions without sacrificing sensitivity in younger age groups makes it a helpful screening tool for a broader range of people. This enables early identification and personalized interventions that take into account individual risk factors [29,46,47].

## 5. Disadvantages/Limitations of Breast Ultrasound in Breast Cancer Screening

Although breast ultrasonography offers certain benefits, it does possess limitations and drawbacks when utilized for breast cancer screening, particularly when contrasted with alternative imaging methods like mammography and magnetic resonance imaging (MRI). Recent studies and papers have highlighted many difficulties linked to breast ultrasonography.

### 5.1. Lower Specificity and Increased False Positive Results

A significant disadvantage of breast ultrasonography is its reduced specificity in comparison to mammography. Although ultrasound is quite effective at identifying abnormalities, it tends to generate a higher number of false positive results. This can result in unnecessary additional tests and possibly invasive procedures like biopsies. The difficulty lies in precisely distinguishing between benign and malignant tumors. Recent research highlights the need to tackle this constraint in order to prevent the potential negative consequences linked to overdiagnosis and treatment [6,36,42].

### 5.2. Operator Dependence and Variability

Breast ultrasonography is highly dependent on the operator’s expertise and experience, resulting in variability and reliance on the operator’s abilities. The proficiency of the technician or radiologist performing the ultrasound examination can influence the quality and accuracy of the results. Recent research highlights the necessity of standardized training and practices to address this constraint and guarantee consistent and dependable outcomes in various healthcare environments [27,48,49].

### 5.3. Low Ability to Detect Microcalcifications

Microcalcifications are very small calcium deposits that can be a sign of early-stage breast cancer [50]. Mammography is highly effective in detecting these microcalcifications, offering a significant benefit in early diagnosis. Nevertheless, breast ultrasonography has limitations in correctly detecting microcalcifications. This constraint is especially noteworthy when considering its function in identifying specific categories of imperceptible, initial-phase breast tumors. Recent research highlights the significance of employing a multimodal strategy, which involves the integration of many imaging methods, in order to achieve a correct breast cancer screening [42,43,44].

### 5.4. Limited Performance in High-Risk or Dense Breast Tissue

Although breast ultrasound may provide benefits for women with thick breast tissue and high-risk patients, new studies have emphasized its limitations in specific situations. Ultrasound may not offer the same level of sensitivity as MRI in persons with highly dense breast tissue or those belonging to high-risk populations. This limitation highlights the significance of customizing screening procedures according to individual risk factors and breast density, guaranteeing that the selected imaging method corresponds to the specific attributes of the patient’s breast tissue [39,51,52,53].

## 6. Advantages of Breast Ultrasound in Breast Cancer Diagnosis

The use of breast ultrasonography is extremely helpful in a comprehensive assessment of breast cancer, providing numerous advantages in the diagnosis process. Recent research and papers emphasize these benefits, emphasizing its dependability, especially when utilized alongside other imaging techniques.

### 6.1. Characterization of Lesions

Breast ultrasound is highly effective in examining breast abnormalities, offering comprehensive data regarding their dimensions, morphology, and internal attributes. This capacity is essential for assessing the probability of malignancy and providing guidance for subsequent diagnostic and therapy choices. Recent studies highlight the exceptional sensitivity of ultrasonography in differentiating between benign and malignant tumors, leading to a more precise and customized diagnostic strategy [16,54,55,56].

### 6.2. Real-Time Imaging and Guided Biopsies

The real-time imaging capacity of breast ultrasound gives a noteworthy advantage in the diagnostic procedure. Clinicians are able to observe abnormalities in a dynamic manner, which helps them accurately focus on specific areas during biopsies. Ultrasound-guided biopsies are precise and minimally invasive techniques that can be performed in real-time to accurately sample worrisome lesions. Recent studies highlight the significance of ultrasound-guided procedures in improving diagnostic precision and minimizing the necessity for more invasive interventions [16,57,58,59].

### 6.3. No Ionizing Radiation

This method’s advantage in screening is also seen in diagnosis—the lack of ionizing radiation. This makes it a safe option for repeated imaging and follow-up assessments, particularly for individuals who may be more sensitive to radiation exposure. Recent studies highlight the importance of considering the cumulative radiation dose in healthcare, making ultrasound a favorable choice for diagnostic evaluations, especially in younger populations or those requiring frequent imaging [16,18,26,60].

### 6.4. Supplementary Imaging in Challenging Cases

Breast ultrasonography is a useful additional imaging technique, especially when the mammography results are unclear or difficult to interpret. Recent research highlights the importance of using it to assess dense breast tissue, offering supplementary information that enhances mammographic findings. Ultrasound is a dependable and supplementary diagnostic tool that enhances the overall sensitivity of the diagnostic process in situations where mammography may produce false negatives or encounter difficulties in visualizing particular lesions [61,62,63].

## 7. Disadvantages/Limitations of Breast Ultrasound in Breast Cancer Diagnosis

Although breast ultrasound is useful in specific areas of breast cancer diagnosis, it does possess significant drawbacks, especially when compared to alternative imaging modalities such as mammography and magnetic resonance imaging (MRI). Recent studies and papers underscore these constraints, highlighting the necessity for a sophisticated and multifaceted approach to diagnosis.

### 7.1. Operator Dependence and Variability

The diagnostic accuracy of breast ultrasonography is susceptible to potential variations due to the operator’s skills and experience. Current studies highlight the importance of implementing standard instruction to address the issue and guarantee consistent and dependable outcomes in various healthcare environments. On the other hand, mammography and MRI are frequently regarded as being more objective and less reliant on the skills of the operator when it comes to interpreting images. This results in a more uniform diagnostic procedure [19,64,65].

### 7.2. Lower Specificity and Increased False Positives

Breast ultrasound is recognized for its higher sensitivity; however, this advantage is sometimes accompanied by less specificity, leading to a higher incidence of false positive results. Recent research indicates that ultrasonography has the potential to detect lesions that first appear suspicious but are ultimately shown to be non-cancerous after additional examination. This limitation can result in unnecessary biopsies and increased patient concern. Mammography, in contrast, is acknowledged for its superior specificity, which enhances the precision in distinguishing between benign and malignant lesions [16,33,66,67,68].

### 7.3. Challenges in Evaluating Dense Breast Tissue

Although breast ultrasound may provide benefits in certain diagnostic situations, it faces difficulties when assessing thick breast tissue. Ultrasound’s efficacy in detecting abnormalities may be diminished in women with thick breasts, hence reducing its sensitivity. Mammography, because of its capacity to penetrate and image thick tissue, continues to be the favored method for screening in such instances. Moreover, MRI is frequently regarded as superior in evaluating the vascularity of breast tissue and offers useful insights, particularly in high-risk groups or situations where other imaging techniques face difficulties due to dense breast tissue [39,69,70].

A concise overview of the advantages and disadvantages associated with using ultrasound for breast cancer screening and diagnosis, along with a summary of its proper use in various aspects of breast cancer screening and diagnosis, is provided in the table below (Table 1).

## 8. ABUS in Breast Cancer Screening and Diagnosis—Advantages and Limitations

ABUS has revealed numerous benefits in its utilization for the detection and diagnosis of breast cancer. One significant benefit is the extensive and uniform coverage it offers. ABUS, in contrast to handheld ultrasound, utilizes an automated machine to methodically examine the entire breast, guaranteeing a consistent imaging method. This characteristic greatly diminishes the possibility of operator-induced variability, resulting in more dependable and replicable outcomes. The technology’s capacity to provide a comprehensive perspective of breast tissue improves its effectiveness in detecting small abnormalities that might be missed in conventional handheld ultrasound examinations [23,71,72].

Furthermore, studies have underscored the efficiency and time-saving benefits of ABUS in comparison to manual ultrasound. The automated process expedites the imaging procedure, making it a more time-efficient screening tool. This efficiency holds particular significance in high-volume screening settings, where ABUS demonstrates the potential to enhance workflow without compromising diagnostic accuracy. The studies emphasize the advantages of ABUS as a complementary screening modality, especially for populations with dense breast tissue or those requiring regular monitoring, affirming its role as a valuable addition to the armamentarium of breast cancer screening and diagnosis [24,71,73].

While automatic breast ultrasound (ABUS) presents promising advancements in breast cancer screening, it is not without limitations and disadvantages. One notable limitation lies in its potential to generate a high number of false positives. The automated nature of ABUS may result in the detection of benign lesions that could be identified as suspicious, leading to unnecessary anxiety for patients and additional follow-up procedures, including biopsies. This phenomenon may be attributed to the sensitive nature of the technology, emphasizing the importance of striking a balance between sensitivity and specificity to avoid overdiagnosis and overtreatment [71].

Another drawback of ABUS pertains to its relatively limited ability to detect microcalcifications. Mammography excels in visualizing these calcifications, providing crucial information for early detection. ABUS, primarily relying on sound waves, may not offer the same level of sensitivity in identifying these microcalcifications. This limitation underscores the need for a multimodal approach to breast cancer screening, where ABUS complements rather than replaces established methods like mammography. While ABUS holds promise, addressing these limitations is crucial for its optimal integration into comprehensive breast cancer screening programs [31,71].

The following table provides a concise overview and quick comparison between ABUS and handheld breast ultrasound, as well as the key advantages and considerations for each modality in breast imaging, as found in the cited studies. Additionally, it highlights the unique strengths and limitations of breast ultrasound compared to mammography, serving as a comprehensive guide in understanding the capabilities and limitations of each imaging modality in breast cancer screening and diagnosis (Table 2).

**Figure 3:**
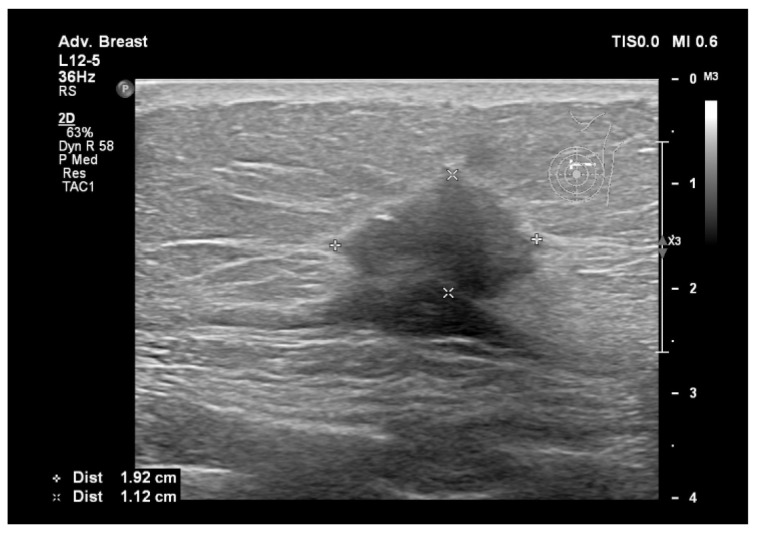
Breast lesion detected using ultrasound, shows a hypoechogenic solid nodule, with spiculations and ill-defined margins.

**Figure 4:**
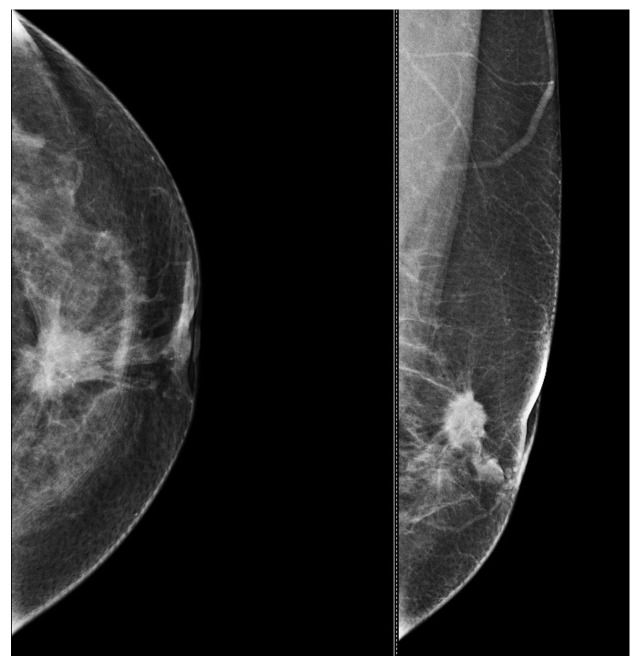
Mammography of the left breast shows a spiculated and ill-defined lesion, that associates skin retraction.

**Figure 5:**
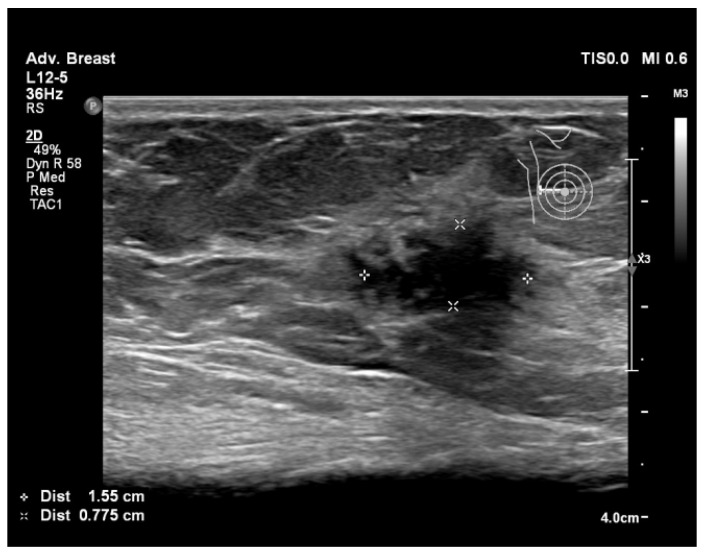
Breast lesion detected using ultrasound, shows a hypoechogenic solid nodule, with spiculations and ill-defined margins.

**Figure 6:**
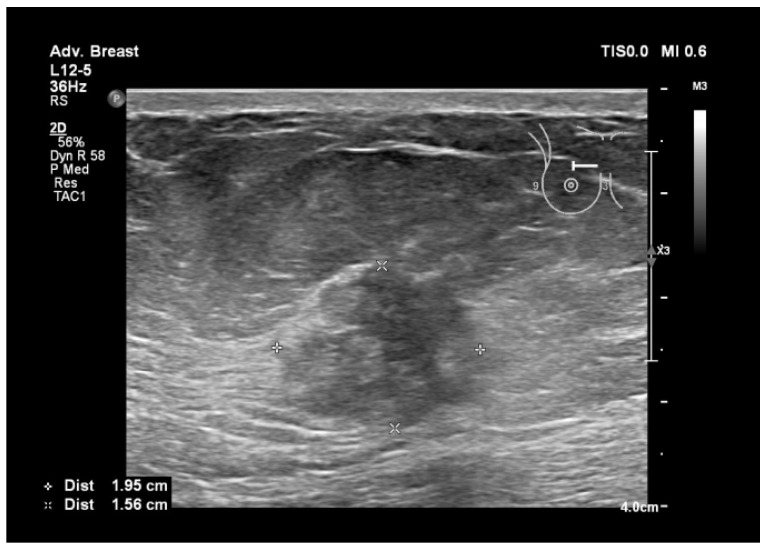
Breast lesion detected using ultrasound, shows a heterogenous, hypoechogenic solid nodule, with spiculations and ill-defined margins.

**Figure 7:**
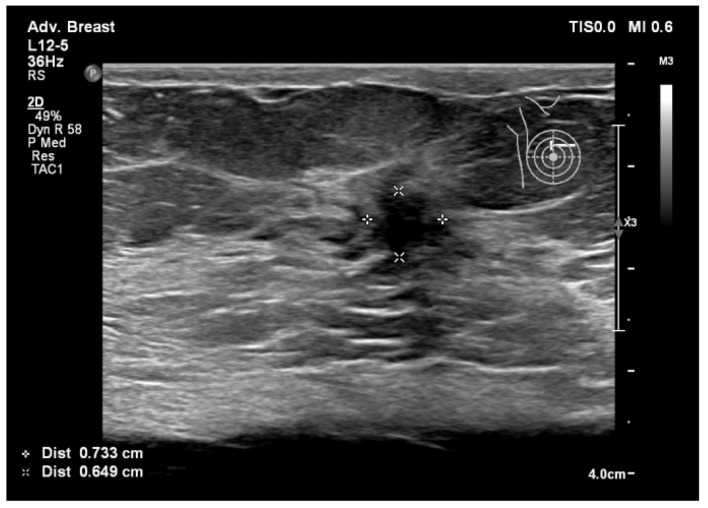
Breast lesion detected using ultrasound, shows a hypoechogenic solid nodule, with spiculations and ill-defined margins.

**Figure 8:**
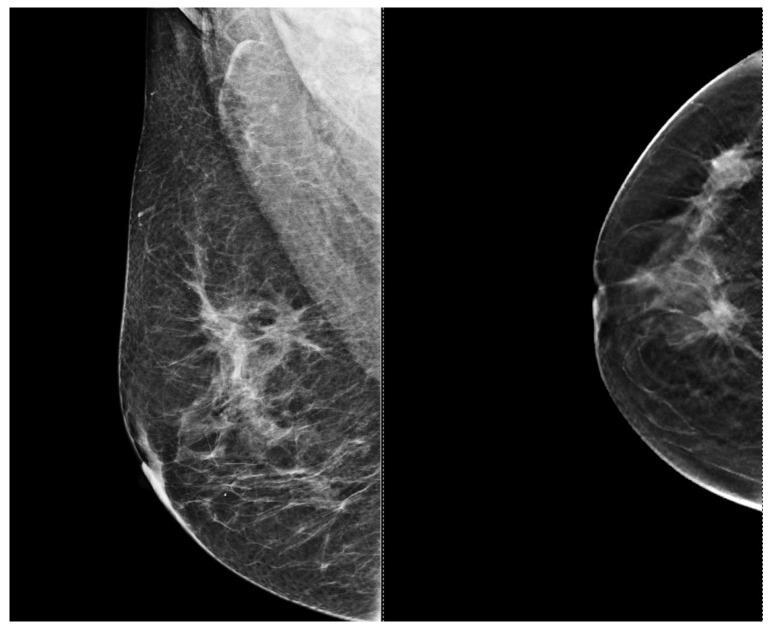
Right mammography shows three spiculated and ill-defined lesions, with malignant characteristics.

To assist in understanding the differences between breast ultrasonography and mammography, we included a few images from our personal archive (Victor Babes Hospital of Infectious Diseases and Pulmonology, Timisoara). All photos were taken with the patients’ informed consent (Figure 3, Figure 4, Figure 5, Figure 6, Figure 7 and Figure 8).

Figure 3 and Figure 4, same suspicious lesion seen both on ultrasound and on mammography in the left breast, presenting ill-defined margins and spiculations on both imaging methods.

Figure 5, Figure 6, Figure 7 and Figure 8, three suspicious lesions seen with ultrasound on the right breast, with the same malignant characteristics on the mammography (spiculations, ill-defined margins).

The following table (Table 3) shows the advantages of breast ultrasound for the evaluation of the breast tissue.

## 9. Types of Lesions Found by Screening with Breast Ultrasound

During breast ultrasonography examinations, incidental, non-glandular lesions may be discovered, complicating the diagnosis. Cysts, fibroadenomas, lipomas, lymph nodes, and benign tumors are among the abnormalities that may be discovered incidentally. While these lesions are often non-glandular and may not be directly related to the primary reason for the ultrasound, they require careful investigation to ensure an accurate diagnosis and proper management. Cysts are among the most common accidental discoveries during breast ultrasonography. These fluid-filled sacs might vary in size and shape, but they are usually benign. Fibroadenomas, or benign solid masses made up of glandular and fibrous tissue, are another typical incidental observation. While fibroadenomas are normally innocuous, they may need further investigation to rule out malignancy, especially in some clinical scenarios.

Lipomas, which are benign adipose tumors, may also be discovered during breast ultrasonography. These lesions usually appear as well-defined, hypoechoic masses and are normally harmless, necessitating no further treatment unless symptomatic or causing discomfort. Lymph nodes may also be seen inadvertently during a breast ultrasound. While lymphadenopathy may arouse worries about cancer, the development of swollen lymph nodes could be a result of inflammation or infection rather than metastatic illness.

Radiologists must carefully capture and characterize these incidental discoveries to ensure proper follow-up and therapy. Depending on the incidental lesion’s characteristics as well as the patient’s clinical history and risk factors, additional diagnostic procedures such as mammography, MRI, or biopsy may be recommended. Clear communication between the referring physician and the patient is critical for guiding further examination and ensuring appropriate patient care [74,75].

## 10. Conclusions

Breast ultrasound has potential as an independent method for screening breast cancer. However, some considerations need to be taken into account, including its capacity to identify specific abnormalities, reliance on the operator’s skills, restricted range of vision, and difficulties in conducting screenings on a wide scale. Additional investigation and rigorous clinical studies are necessary to determine the efficacy and constraints of using it as a main screening tool. The choice to implement it on a large scale depends on a thorough assessment across various demographics.

The evolving field of breast ultrasound in cancer screening and diagnosis involves ongoing research to explore new technologies, including the integration of artificial intelligence to standardize interpretation and enhance diagnostic accuracy. Collaborative efforts are essential to address technical challenges, refine imaging protocols, and position breast ultrasound as a reliable and feasible option, particularly in resource-constrained environments. Despite its acknowledged advantages, recognizing limitations such as operator dependence and challenges in detecting microcalcifications remains vital. Beyond screening, breast ultrasound emerges as a crucial component in multimodal breast cancer management, contributing to preoperative staging, treatment response assessment, and post-treatment surveillance. While not replacing mammography entirely, the distinct advantages of breast ultrasound, including enhanced sensitivity, safety, versatility, accessibility, and suitability for specific populations, establish it as a reliable standalone screening method. Integrating breast ultrasound into comprehensive strategies, especially for populations with unique risk profiles, promotes more effective and personalized breast cancer detection programs.

ABUS emerges as a promising technology in breast cancer screening, offering advantages in standardized imaging and enhanced sensitivity, particularly in populations with dense breast tissue. While ABUS demonstrates notable strengths, the discussion underscores the importance of its complementary role alongside handheld ultrasound, as the two modalities collectively contribute to a more comprehensive and accurate approach to breast cancer screening and diagnosis.

Finally, implementing ultrasound for breast cancer screening and diagnosis in low- and middle-income countries is crucial due to its affordability, portability, and safety. Unlike mammography or MRI, ultrasound requires fewer resources and infrastructure, making it more accessible, especially in rural areas. Its radiation-free nature also ensures safety during repeated screenings, vital for younger populations. Moreover, ultrasound’s real-time imaging aids in early detection, facilitating prompt intervention. By integrating ultrasound into healthcare systems, these countries can enhance early detection rates, reduce mortality, and alleviate strain on healthcare resources. Thus, ultrasound emerges as a vital tool in combating breast cancer in resource-limited settings.

## 11. Limitations of the Study

An obstacle we encountered was the heterogeneity of the chosen research, encompassing variations in their design, participants, and techniques. The presence of heterogeneity in the data may impede the generalizability of the findings to certain patient cohorts or healthcare environments.

A limitation of the current body of research on ultrasound for breast cancer screening and diagnosis is the lack of consistency in reporting the specific types of breast neoplasia studied. Not all studies provide clear categorization of the types of breast lesions or cancers examined, which hinders the ability to draw comprehensive conclusions about the effectiveness of ultrasound across different malignancies. Additionally, another limitation lies in the absence of racial demographic data in some studies. Understanding the racial or ethnic aspects of the women involved in these studies is crucial for assessing the generalizability of the findings and ensuring equitable access to effective screening and diagnostic methods across diverse populations. Thus, the variability in reporting both the types of breast neoplasia and racial demographics underscores the need for more standardized and inclusive research practices in this field.

Another limitation from our point of view is the actual dynamic nature of both ultrasound technology and clinical guidelines. The field of breast cancer screening is continuously evolving, with advancements in ultrasound technology and changes in recommended practices. A literature review may face challenges in keeping pace with these developments, potentially overlooking recent studies or failing to capture shifts in the standard of care. This limitation underscores the need for frequent updates and consideration of the temporal context when interpreting the findings of the review.

## 12. Future Directions

These limitations present avenues for future research to enhance the understanding and applicability of ultrasound in breast cancer screening and diagnosis. Firstly, future studies should aim to provide more detailed categorization of the types of breast neoplasia studied, allowing for a nuanced analysis of ultrasound’s efficacy across different malignancies. This would enable researchers to identify specific subtypes of breast cancer where ultrasound may be particularly beneficial, guiding more targeted screening and diagnostic approaches. Additionally, incorporating racial demographic data into study designs is crucial for ensuring the inclusivity and generalizability of findings. By addressing these limitations, future studies can contribute to a more comprehensive understanding of ultrasound’s role in breast cancer detection and management, ultimately improving outcomes for diverse patient populations.

Another important future direction would be to determine the accuracy of using two distinct probes for breast cancer screening. Studies show that the use of high-frequency probes (>15 MHz) in breast imaging constitutes a considerable advancement, particularly in assessing the vascularization of breast lesions. The American College of Radiology’s recommendation for the availability of two multi-frequency linear probes emphasizes the significance of taking a nuanced approach to breast examinations. The first probe, which operates at frequencies ranging from 7.5 to 14 MHz, is critical for examining deeper layers of breast tissue. This lower frequency range allows for more penetration and comprehensive evaluation of components such as the muscle plane, fascia, and retromammary layer. Furthermore, it is essential for the assessment of large lesions, allowing radiologists to gain insights into their form and spatial distribution within the breast tissue. The second probe, with an upper frequency range of 15 to 24 MHz, gives better resolution, especially when scanning superficial planes. This higher frequency range improves the imaging of minute anatomical details and microvascular structures, providing crucial information on the vascularization patterns of breast diseases. Radiologists can gain critical diagnostic information by methodically examining the vascular architecture of lesions in the superficial layers, which aids in characterization and treatment decisions. Radiologists could obtain a full evaluation of breast lesions by using both probes in the breast examinations, which include both deep and superficial structures. This comprehensive approach improves the diagnostic accuracy while also allowing for more individualized therapy planning and patient care. It also emphasizes the necessity of exploiting technology breakthroughs in breast imaging to improve patient care and results in the field of breast cancer detection and treatment [76].

Some other future directions regarding the use of breast ultrasound for breast cancer screening may include the potential assessment of integrating AI algorithms with breast ultrasound, in order to improve the accuracy in detecting breast cancer lesions, and to investigate the development of AI-based tools to assist radiologists in interpreting ultrasound images and differentiating between benign and malignant findings.

Another direction may include an exploration of the feasibility of using portable and point-of-care ultrasound devices for breast cancer screening in resource-limited settings, enabling wider accessibility and early detection in diverse populations.

Future studies on ABUS should assess its long-term efficacy and cost-effectiveness in diverse populations through large-scale, multicenter clinical trials with extended follow-up. Integrating artificial intelligence to enhance ABUS accuracy, exploring point-of-care applications, and evaluating patient preferences would inform practical implementation. Assessing ABUS feasibility in resource-constrained environments and its impact on reducing health disparities is crucial. This research aims to shape evidence-based guidelines and optimize ABUS integration into routine breast cancer screening and diagnosis protocols.

Adjusting technical parameters such as the Pulse Repetition Frequency (PRF) is critical in Doppler assessments of breast lesions, particularly for improving sensitivity to low-flow circumstances typically found in breast tissue. Lower PRF settings are favored in such circumstances because they facilitate the detection of slow-flowing arteries within lesions. Furthermore, a gel stand-off pad is often used to improve acoustic interaction between the transducer and the skin surface. This technique reduces artifacts and enhances the observation of blood flow patterns within breast lesions, hence minimizing signal attenuation and improving overall Doppler picture quality. Future studies in Doppler evaluation should investigate enhanced Doppler imaging techniques, such as power Doppler or color Doppler, to better define vascular patterns and improve diagnostic accuracy [77,78]. Furthermore, research efforts should concentrate on the creation of automated algorithms or artificial intelligence-based tools to help radiologists analyze Doppler pictures and measure blood flow characteristics more effectively. Furthermore, prospective studies could look into the clinical value of Doppler imaging as a non-invasive biomarker for predicting tumor aggressiveness, responsiveness to therapy, and overall prognosis in breast cancer patients. Such trials show promise for expanding the role of Doppler assessment in breast lesion detection and management, potentially leading to better patient outcomes and clinical decision-making.

Last, but not least, another direction of study may explore the role of breast ultrasound as an integral component of multimodal breast cancer management, including its use in preoperative staging, treatment response assessment, and post-treatment surveillance.

## Figures and Tables

**Table 1:** Comprehensive overview: ultrasound in breast cancer screening and diagnosis.

Aspect	Ultrasound in Breast Cancer Screening and Diagnosis
Advantages	- No ionizing radiation: safe with no radiation exposure - Suitability for repeated screenings: safe for frequent tests - Preferred for younger populations: safe and preferred - Psychological comfort: non-invasive and reduces anxiety - Enhanced sensitivity in dense tissue: effective detection - Real-time imaging and guided biopsies: precise procedures - Versatile and dynamic imaging: useful for various conditions - Accessibility and cost-effectiveness: affordable and portable - Application in high-risk populations: effective screening
Disadvantages	- Lower specificity: potential for more false positives - Operator dependence: results vary with operator’s experience - Inability to detect microcalcifications: limited in early detection - Challenges in large population screening: logistically challenging - Limited performance in dense tissue: sensitivity may be compromised - Challenges in screening high-risk populations: lower sensitivity - Limited ability to detect microcalcifications: operator-dependent
Applications	- Screening: Advantages: enhanced sensitivity, especially in dense tissue. Considerations: operator-dependent, potential for false positives.
	- Diagnosis: Advantages: lesion characterization, real-time imaging, guided biopsies. Considerations: limited microcalcification detection, operator dependence.
	- Multimodal Management: Advantages: comprehensive approach when integrated with other techniques. Considerations: coordination between modalities required.
	- Point-of-care applications: Advantages: portable, adaptable for quick assessments. Considerations: may not replace comprehensive exams in all cases.
	- Resource-constrained environments: Advantages: cost-effective, accessible. Considerations: limited by equipment and expertise availability.

**Table 2:** Comparison of ABUS and handheld breast ultrasound: advantages, considerations, and a concise overview compared to mammography.

Feature	ABUS	Handheld Ultrasound
Process of the Technique	Automated scanning providing standardized images	Operator-dependent, manual control over scanning areas
Coverage	Comprehensive coverage reducing operator variability	Limited coverage, dependent on operator’s skill and experience
Operator Dependence	Reduced operator dependence, minimizing variability	High dependence on operator’s skills, potential variability
Detection Accuracy	High sensitivity with systematic approach	Detection accuracy varies based on operator’s proficiency
Limitations	May generate more false positives, limited microcalcification detection	Operator-dependent, potential coverage and reproducibility limitations, limited microcalcification detection
**Aspect**	**Breast Ultrasound**	**Mammography**
Advantages	- Absence of ionizing radiation - Suitable for repeated screenings and younger patients - Real-time imaging enables dynamic evaluation and precise biopsies - Comfortable for patients	- High specificity, fewer false positive results - Superior microcalcification detection, aiding early detection - Established effectiveness and reliability as primary screening tool - Well-established infrastructure and standardized protocols
Disadvantages	- Lower specificity, increased false positives - Operator dependence and variability impacting consistency and accuracy - Limited microcalcification detection, challenges in dense breast tissue	- Utilizes ionizing radiation, potential risks over repeated screenings - Limited performance in dense breast tissue, potentially reduced sensitivity - Less dynamic imaging, limited ability to evaluate abnormalities in real time - May cause discomfort during procedure, affecting patient compliance

**Table 3:** Overall advantages of breast ultrasound for breast tissue evaluation in a quantified form.

Research Focus	Sample Characteristics
Overall advantages of breast ultrasound for breast tissue evaluation	Individuals requiring regular screenings
	Those with family history of breast cancer or genetic predisposition
	Younger patients, particularly females below 40 years old
	Patients seeking comfort and decreased fear during imaging
Advantages of breast ultrasound in breast cancer screening	Women with dense breast tissue
	Versatile and dynamic real-time imaging
	Accessibility and cost-effectiveness
	Application in high-risk populations and younger women
Disadvantages/limitations of breast ultrasound in breast cancer screening	Increased false positive results
	Operator dependence and variability
	Limited ability to detect microcalcifications
	Limited performance in high-risk or dense breast tissue
Advantages of breast ultrasound in breast cancer diagnosis	Characterization of lesions
	Real-time imaging and guided biopsies
	No ionizing radiation
	Supplementary imaging in challenging cases
Disadvantages/limitations of breast ultrasound in breast cancer diagnosis	Operator dependence and variability
	Lower specificity and increased false positives
	Challenges in evaluated dense breast tissue
ABUS in breast cancer screening and diagnosis	Automated and uniform coverage
	Efficiency and time-saving benefits
	High false positive rate
	Limited ability to detect microcalcifications
	Requires a multimodal approach for comprehensive screening

## Data Availability

Data are contained within the article.
